# A Contemporary Review of Neurological Sequelae of COVID-19

**DOI:** 10.3389/fneur.2020.00640

**Published:** 2020-06-23

**Authors:** Brian Fiani, Claudia Covarrubias, Aditi Desai, Manraj Sekhon, Ryan Jarrah

**Affiliations:** ^1^Desert Regional Medical Center, Palm Springs, CA, United States; ^2^Universidad Anáhuac Querétaro, Santiago de Querétaro, Querétaro, Mexico; ^3^Institute of Medical Science, University of Toronto, Toronto, ON, Canada; ^4^Oakland University William Beaumont School of Medicine, Rochester, MI, United States; ^5^College of Arts and Sciences, University of Michigan-Flint, Flint, MI, United States

**Keywords:** COVID 19, coronavirus, neurological sequelae, neurological symptoms, neuroinfection

## Abstract

Coronavirus 2019 (COVID-19) is currently the center of what has become a public health crisis. While the virus is well-known for its trademark effects on respiratory function, neurological damage has been reported to affect a considerable proportion of severe cases. To characterize the neuro-invasive potential of this disease, a contemporary review of COVID-19 and its neurological sequelae was conducted using the limited, but growing, literature that is available. These neurological squeal are based on the manifestations that the virus has on normal central and peripheral nervous system function. The authors present the virology of the SARS-CoV-2 agent by analyzing its classification as an enveloped, positive-stranded RNA virus. A comprehensive timeline is then presented, indicating the progression of the disease as a public health threat. Furthermore, underlying chronic neurological conditions potentially lead to more adverse cases of COVID-19. SARS-CoV-2 may reach ACE2 receptors on neuronal tissue through mode of the general circulation. The CNS may also be susceptible to an immune response where a “cytokine storm” can manifest into neural injury. Histological evidence is provided, while symptoms such as headache and vertigo are highlighted as CNS manifestations of COVID-19. Treatment of these symptoms is addressed with paracetamol being recommended as a possible, but not conclusive, treatment to some CNS symptoms. The authors then discuss the peripheral nervous system sequelae and COVID's impact on causing chemosensory dysfunction starting with viral attack on olfactory sensory neurons and cells types within the lining of the nose. Histological evidence is also provided while symptoms such as anosmia and ageusia are characterized as PNS manifestations. Possible treatment options for these symptoms are then addressed as a major limitation, as anecdotal, and not conclusive evidence can be made. Finally, preventive measures of the neurological sequelae are addressed using a multidirectional approach. Postmortem examinations of the brains of COVID-19 patients are suggested as being a possible key to formulating new understandings of its neuropathology. Lastly, the authors suggest a more comprehensive neurological follow-up of recovered patients, in order to better characterize the neurological sequelae of this illness.

## Introduction

### Epidemiology

The emergence of the coronavirus and its viral agent, severe respiratory syndrome coronavirus 2 (SARS-CoV-2), has derived into one of the most unprecedented and considerable pandemics of the modern era. The third zoonotic coronavirus this century, its pathogenic nature and large scale adverse medical effects has cemented its status as one of history's greatest public health threats. This virus was first isolated in December 2019 after many patients in Wuhan, China, were diagnosed with pneumonia of unknown etiology (1–3). Following its global dispersion, the World Health Organization (WHO) declared the coronavirus as a pandemic due to its rapidly transmissible nature, increasing mortality rate, and limited treatment options. Currently, there is an ongoing outbreak as the number of COVID-19 cases exponentially increases with a reported low to moderate mortality rate internationally. As of late May 2020, there are over 5.7 million cases confirmed worldwide dispersed throughout 212 countries and territories, including almost 353,000 deaths (1, 2).

Until recently, COVID-19 was investigated as primarily a respiratory illness. However, as the number of cases continue to grow, more reports are starting to show that the virus is capable of not only lung damage, but also neurological damage. In a case series from Wuhan, neurological symptoms were noticed in 36.4% of patients with severe infections (4). This notable proportion of cases presents a question on what neurological symptoms are manifested along with how the virus manages to present them. As of May 1st, 2020, remdesivir has been approved for some emergency treatment usage. However, no other clinically approved vaccine or specific antiviral treatments has provided conclusive relief to COVID-19 and its physiological manifestations. Therefore, understanding the clinical impact of SARS-CoV-2, along with its underlying mechanisms is of great importance to promote the development of effective, preventative, and therapeutic counteragents. Herein, we will characterize the virus, along with its neurological impact, through a thorough a review of the literature to date.

### Virology

Coronaviruses (CoVs; family *Coronaviridae*, subfamily *Coronavirinae*) circulate in a diverse array of mammalian and avian reservoirs. CoVs are classified into four genera and are enveloped, positive-stranded, RNA viruses known to have large genomes susceptible to recombination and mutations that lead to the emergence of novel viruses. They can cause respiratory, enteric, hepatic, and neurological diseases (5–7). There are still some inconsistencies related to the origin of SARS-CoV-2, with research evidence suggesting that it could be from similar origins of its viral relatives. Both betacoronaviruses, severe acute respiratory syndrome coronavirus (SARS-CoV) and the Middle East respiratory syndrome coronavirus (MERS-CoV), had originated in bats, and it is likely that SARS-CoV-2 did as well from an unknown intermediate host (3, 5, 6).

The estimated reproduction number (R_0_) for SARS-CoV-2 ranges from 2.2 to 5.5 (3, 8, 9), meaning one person can potentially transmit the disease to 5–6 people, making it highly transmissible. Additionally, SARS-CoV-2 research has revealed specific physiochemical and thermal sensitivities. Properties of SARS-CoV-2 can be inactivated by UV light or at a temperature of 56°C for 30 min. Disinfectants such as diethyl ether, 75% ethanol, chlorine, peracetic acid, and chloroform destabilize its functional integrity (8). The virus has the longest viability on stainless steel and plastic surfaces and can be detected up to 72 h after initial contact to these surfaces (8). Genomic studies of the virus have revealed that it mutated to produce two variants, L and S. The L-type was more prevalent during early stages of the outbreak and can replicate faster while being more transmissible. The S-type is considered an older and milder variant (8).

### Timeline: From History to Today

**Figure 1:**
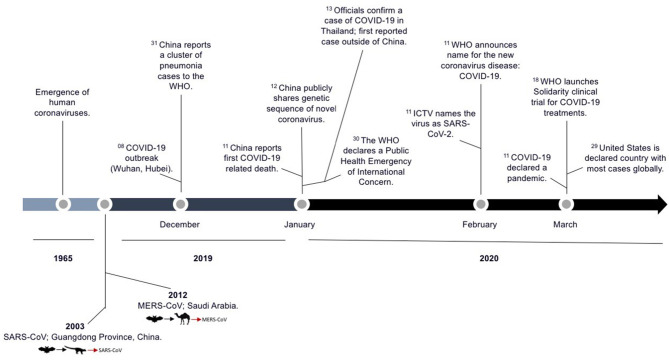
Timeline of the SARS-CoV-2 outbreak. Significant epidemiological events and scientific advances during this time period are highlighted and adapted from the WHO and recently published data (1–3, 10). The superscript number adjacent to the text indicate the calendar date of each event described.

The first human coronavirus was detected by Tyrrell and Bynoe in the 1960s in human embryonic tracheal organ cultures obtained from the respiratory tract of an adult with a common cold. After performing electron microscopy on the samples of this new group of viruses, the morphology denoted a crown-like appearance of the surface projections and thus was named coronavirus (3, 10). Since then, SARS-CoV-2 is the seventh coronavirus that is known to infect humans. The first pandemic of the 21st century was caused by SARS-CoV followed by MERS-CoV. In 2002, the SARS-CoV emerged in Guangdong Province, China, spreading to 37 countries, and its subsequent global epidemic was associated with 8,096 cases and 774 deaths. A decade later, the MERS-CoV spread to 27 countries, causing 2,494 infected cases and 858 deaths worldwide (Figure 1) (2, 6).

## Susceptibility

According to the Centers for Disease Control and Prevention, risk of susceptibility to COVID-19 can be stratified into risk of exposure and risk of severe illness (11). Increased risk of exposure to SARS-CoV-2 has been shown to occur in places with ongoing community spread, such as between healthcare workers, close contacts of persons with COVID-19, and travelers from affected international locations. Of those infected, increased severity of illness can be seen more commonly in people aged 65 years and older, those living in a nursing home or long-term care facility, and in people with underlying medical conditions such as hypertension, chronic lung disease, moderate to severe asthma, cardiac disease, immunocompromised states (i.e., cancer, smoking, HIV or AIDS, steroids), BMI > 40, diabetes, chronic kidney disease, and liver disease (11–16). Moreover, The Chinese Center for Disease Control and Prevention similarly reported from one of their largest studies of 72,314 cases that high case-fatality rates came from those with similar comorbidities: cardiovascular disease (10.5%), diabetes (7.3%), chronic respiratory disease (6.3%), hypertension (6%), and cancer (5.6%) (17). In addition, those with cerebrovascular disease alongside coronary artery disease had a higher relative risk of acquiring the pathogen more severely than those without this comorbidity (18). Finally, and most applicable to the area of interest, those with chronic neurological pathologies present an added risk of developing a more severe case of COVID-19 (19). Neurological or neurodevelopment conditions (such as cerebral palsy, epilepsy, stroke, intellectual disabilities, spinal cord injury, or muscular dystrophy), present additional risk factors that increase susceptibility to a severe course of COVID-19 through mechanisms that add physiological stress (19). The current epidemiology demonstrates the impact SARS-CoV-2 can have on the health and well-being of people with underlying health conditions, highlighting its potential effects on multiple organ systems in the body. However, due to this virus being relatively new, not all risk factors have been identified adding limitation to this understanding.

## Neurological Impact

### Central Nervous System

With much being said about SARS CoV-2's hallmark impact on respiratory function, gradual advances in research have found properties of neuroinvasive potential, particularly in the invasion of the central nervous system (CNS). It is widely understood that the mode of transmission of COVID-19 is through direct contact or respiratory droplets from an infected individual. However, indirect contact through fomites can also act as a vehicle of transmission. Fomites are inanimate surfaces that contain the virus (through direct contact), and based on the viability of the virus on to an inanimate surface, it can lead to the rampant spread onto another host. As soon as the virus enters the body, its effects are highly dependent on its viral structure. The viral structure of COVID-19 is contained within the nucleocapsid which is surrounded by a nuclear envelope. The nuclear envelope is derived from the host cell's membrane and embedded within that membrane are glycoprotein spikes, known as S-proteins (20). It is these S-protein spikes that allow the cell to attach to a new cell and infect it (20). To carry through its function, S-proteins contain an S1/S2 activation cleavage site that is activated by the serine endoprotease, furin (21). Furin is a key protein that can cause normal and abnormal physiological conditions. It is important neurologically because it is responsible for activating neural growth factors that are key to homeostasis (22). However, in the case of SARS-CoV-2, furin plays the role of a viral activator. This activation occurs through furin's enzymatic activity, which allows for the irreversible cleavage of precursor proteins to enter their biologically active state (21). With regards to SARS-CoV-2, furin autocleavage helps the S-protein's subunits to separate, and allow for the virus to break open and enter host cells (21). This is done after the S-proteins have interacted with the cell surface receptor, angiotensin-converting enzyme 2, or ACE2 (21). The ACE2 receptor is present along multiple cell organs such as the heart, kidney, lunges, while also being found in both the central and peripheral nervous system. This makes it a valuable target in understanding the neural-potential of the SARS-CoV-2 antigen.

The pathophysiology of neural infections in the CNS from COVID-19 is a phenomenon that is still being characterized. Previous studies have shown that SARS-CoV-2 may reach ACE2 receptors on neuronal tissue through mode of the general circulation. Viremia causes the virus to pass into the cerebral circulation and reach the brain to induce neurotropic effects. The virus may also reach the brain through routes of the nasal cavity, specifically as the virus passes through the cribriform plate. Upon entry into the neural cavity, the virus will encounter ACE2 receptors located on the endothelial lining between the blood capillaries and the brain (23). The virus may also encounter the expression of ACE2 receptors over glial cells and neurons, allowing for multiple sites of invasion. The interaction of the glycoproteins of SARS-CoV-2 to the ACE2 receptor can cause subsequent cycles of viral budding, allowing for neuronal damage to manifest in neural tissues and the blood-brain-barrier (BBB) (23). The breaking of the BBB may allow for cerebral edema, subsequently compressing the brainstem and compromising involuntary respiratory activity (24). This cerebral edema may also be caused by apoptosis of brain cells following elevated intracranial pressure and therefore remains a theoretical concern of the virus. Evidently, patients exhibiting acute SARS-CoV illnesses have presented the virus in cerebrospinal fluid analyses (23). The cerebrospinal fluid (CSF) is a bodily fluid that surrounds the brain and serves an array of functions ranging from acting as a shock absorber from neurological trauma to circulating nutrients around the CNS. Its role in homeostasis and neurological metabolism has made it a great reporter of the neurological environment; therefore, it can be clinically used to detect infectious agents and diseases (25). Furthermore, viral implications on the immune system could induce indirect effects on the CNS. The breakage in the blood-brain barrier could leave an immune response of cytokines (particularly IL-6) becoming overly abundant along infected tissue of the brain (26). This leads to a hypercoagulable state in what is known as a “cytokine storm.” Depending on the severity of the case, this could leave COVID-19 patients at risk of developing acute necrotizing encephalopathy (ANE) and hemorrhages (27). Most recently, a third theory discusses how neural injury is manifested from COVID-19 as a result of a cascade effect following respiratory stress. A loss of oxygen due to lung damage can subsequently result in multisystem organ failure leading to a cascade effect that results in neural injury (28). Ultimately, what these three theories provide are multiple mechanisms through which neural manifestations of COVID-19 can result. These theories also highlight the limitations in addressing neurological manifestations in COVID-19, as research remains ongoing.

A recent case of a female patient in Detroit, Michigan showed the possible long-term impact of neurological manifestations of COVID-19 (27) while also presenting some histological significance. This patient was jointly diagnosed with COVID-19 and ANE due to possible subsequent neural injury. CT scans of her brain revealed symmetrical tissue damage of the thalamus while her MRI also showed damage to the thalamus along with the cerebral cortex and the brain tissue beneath its gyri (folds within the cerebral cortex) (27). Histological significance is evident in her CT image as hypodense areas are present, indicating tissue damage had occurred. This decrease in density is a result of cerebral edema, when excess fluid floods brain tissue after neural injury, a possible consequence of severe CNS manifestations of COVID-19 (27). As previously stated, the cause of this cerebral edema is possibly through a “cytokine storm,” apoptosis of brain cells, or even from breakage in the BBB. In addition, other severe cases aside from ANE and hemorrhage are prevalent, as the first case of meningitis has been associated with COVID-19 (29). A male patient was found to have this condition after his MRI showed hypersensitivity along the right lateral ventricle, as well as hyperintesnse signal changes in the right mesial temporal lobe and hippocampus (29). He was confirmed to have COVID-19 after SARS-CoV-2 was found in his CSF, however, there was no evidence of cerebral edema like the previous case mentioned (29). Thus, the variability in histological evidence could link theories of pathophysiology regarding COVID-19's impact on the CNS.

While the more severe symptomatic manifestations of the CNS may include ANE, meningitis, and hemorrhage, the more commonly reported CNS symptoms are vertigo, cephalgia, impaired consciousness, seizures, ataxia, and acute cerebrovascular disease (30) (Table 1). In a study published to JAMA Neurology, patients with CNS manifestations most commonly exhibited headache and dizziness (30) as the major symptoms. These manifestations have been reviewed and confirmed by trained neurologists, with most neurological symptoms occurring during the early stages of the illness (30). Laboratory findings of these patients reported lower lymphocyte and platelet counts and higher blood urea nitrogen levels. However, in more mild cases, no significance was found between patients with or without CNS manifestations of COVID-19 (4). Therefore, it was concluded that patients with severe cases were more susceptible to neurological symptoms. Moreover, the more severe cases had higher fibrin degradation product levels (D-dimer), linking these cases to a higher likelihood of cerebrovascular disease (4). Furthermore, recent findings have reported that a portion of patients are developing symptoms of stroke. Specifically, reports coming out of New York health systems have reported that large vessel strokes can be manifested, even in individuals under the age of 50 (31). A large vessel stroke is an interruption of blood flow in one of the larger arteries in the brain. The suspected cause of these strokes are investigated as an immunohematological issue in allowing for blood clotting throughout a patient's body (31).

**Table 1:** Overview of COVID-19's impact on the central and peripheral nervous system, as known to date.

	**CNS Overview of COVID-19**	**PNS Overview of COVID-19**
How is it transferred?	Directly: respiratory droplets & direct contact with an infected individual Indirectly: Fomites/Surfaces	Directly: respiratory droplets & direct contact with an infected individual Indirectly: Fomites/Surfaces
Pathophysiology theories	1. Viral Entry into the Brain 2. Adverse-Immune Responses 3. Respiratory Stress	1.Chemosensory Dysfunction
Histological significance found:	Hypodense areas in CT scans, following positive testing for COVID-19	Falling out of hair-like receptors on olfactory tissue
Main symptoms:	Headache and Vertigo	Anosmia and Ageusia
Major symptoms:	Stroke, Meningitis, ANE, Hemorrhages	Guillain-Barre Syndrome (GBS) and Miller Fisher Syndrome
Treatment/Management/Recovery:	No conclusive treatment: Paracetamol has been seen to provide headache and vertigo relief without worsening COVID-19 symptoms	No conclusive treatment: Nasal Spray being developed for medication delivery

*Limitations are prevalent as a neurological understanding is yet to be fully confirmed*.

Treatment of COVID-19 and its CNS manifestations are still ongoing. However, if the coronavirus is breaching the blood-brain barrier, this could complicate the recovery process (32). The role of the BBB is to serve as the gatekeeper in cerebral passage, therefore medication delivery to the BBB would be difficult because medications are considered as antigens to it, thereby preventing their therapeutic effects (32). Since differences in genetic makeup can increase susceptibility to viral invasion of the BBB, technologies such as CRISPER/CAS9 presents a possible way to target these adverse genetic differences. By targeting specific sequences to engineer candidate risk genes in the BBB, one can assess if BBB permeability is strengthened (33). With regards to the symptomatic prevalence of the disease, hydroxychloroquine has been notably investigated as a possible therapeutic avenue. However, in certain patients, this antimalarial drug may induce adverse neuropsychiatric effects, such as worsening of CNS symptoms like headaches, vertigo, and increased anxiety (30). This makes hydroxychloroquine a less viable treatment for symptoms pertaining to the CNS, and alternative therapies should be considered for patients suffering from these symptoms or from chronic mental instability (22). Furthermore, the NHS recommends paracetamol for CNS-like symptoms such as fevers and headaches due to its reported symptom relief and low likelihood of complications. However, ibuprofen and other NSAIDs are still being evaluated after being ruled out as a therapeutic option (34). They were initially ruled out due to reports that anti-inflammatory drugs may exacerbate COVID-19 symptoms, due to possibly causing a dampened immune response to viral attack (35). These reports have since been rejected by the World Health Organization due to a lack of evidence suggesting that anti-inflammatory treatments are a direct threat to a patient's immunity (34). However, this does not present NSAIDs as a potential therapy, especially when symptoms become more complicated. With regards to blocking viral activity and ultimately CNS manifestations, furin inhibitors have also been investigated as a possible treatment by preventing the activation of the S-proteins on the viral surface (30). However, with furin's important role in normal physiology, researchers are evaluating whether a molecule could be secreted to separate its activity from the virus. Unfortunately, there are still considerable limitations in finding a viable treatment for COVID-19's CNS manifestations, as clinical trials will continue until a vaccine becomes readily available.

### Peripheral Nervous System Involvement

COVID-19, like its pathogenic relatives, could also specifically compromise the activity of the peripheral nervous system. Just like the CNS, PNS manifestations arise following direct contact through respiratory droplets or indirect contact through fomites. However, as an individual starts to touch mucosal membranes, such as their eyes, nose, or mouth, it presents a portal of entry for the virus to disrupt neurological sensations that pertain to these sites. Moreover, just like the CNS manifestations, furin's endoproteolytic activity is maintained in order to activate the virus following its interactions with ACE2 receptors. These consistencies are not just present within the nervous system but along all physiological systems that get affected through COVID-19. However, COVID-19, has a peripheral neural involvement that occurs when ACE2 receptors are interacting with the viral agent. These ACE2 receptors have been found along the tongue, mouth, and nasal passage showing the possibility of the virus targeting multiple neurons and influencing those senses tremendously (36).

**Figure 2:**
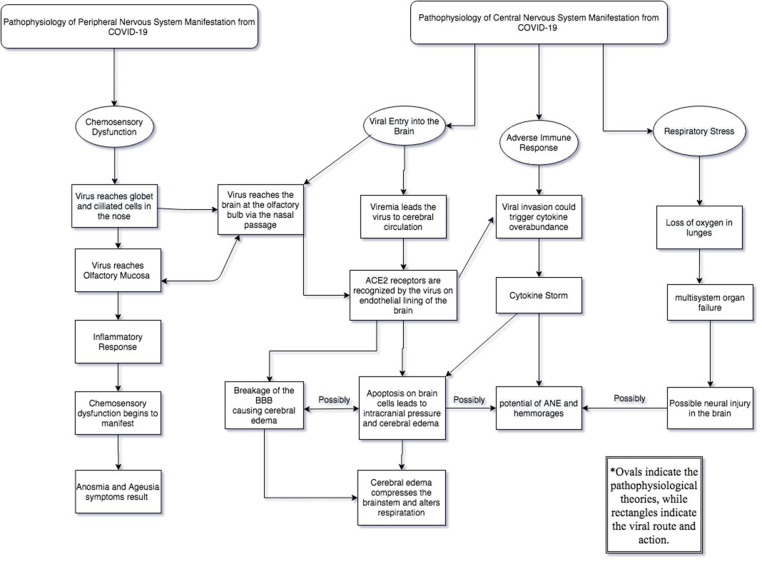
The pathway of the virus in the central and peripheral nervous system which are connected when the virus reaches the olfactory bulb.

It was recently discovered that two cell types in the nose are the most likely initial infection points for viral entry into the PNS. These two cell types are called goblet cells and ciliated cells (37). Goblet cells are epithelial cells that can produce mucus on the surface of the mucosal membrane, thereby lubricating the epithelium and protecting it against foreign invaders (38). They are found in several physiological systems, including the respiratory system, consequently leading to their involvement in the pathophysiologies of several respiratory diseases such as COVID-19 (38). On the other hand, ciliated cells are another cell type that is known for having tiny hair-like projections anchored along the lining of the epithelial respiratory tract. Ciliated cells aid in mucus transport and stopping foreign bacteria from reaching the lunges (39). These two cell types are related in the sense that they both contain high concentrations of the ACE2 receptor that allows SARS-CoV-2 to enter host cells (38). In addition, they contain high concentrations of another protease called TMPRSS2 (38). TMPRSS2 is a cell surface protease that can have cumulative effects with furin to promote SARS-CoV-2 activation and entry (40). To understand what types of cells are infected following initial viral entry, it is important to understand that the sense of smell and taste are closely associated with one another. Both rely on the same type of sensory cells, called chemoreceptors, which are activated when they meet certain types of chemical stimuli. SARS-CoV-2 could disrupt these chemoreceptors by reaching the olfactory mucosa (which contains epithelium cells, blood vessels, and axons from olfactory neurons) and initiate an inflammatory response (24). This area is joined with the olfactory bulb by the cribriform plate, which allows for the transmission of olfaction senses at the base of the frontal lobe (41). Here, neurons can be infected while endangering deeper cerebral tissue (24). This highlights the linkage between the CNS and PNS during the viral invasion through the nasal cavity (Figure 2). Moreover, should defects in respiration be considerable, then PNS chemoreceptic dysfunction may be presented due to the proximity of sensory neurons in the olfactory bulb to deeper brain tissue (42).

Histological significance pertaining to the PNS involvement was found following analysis of infected olfactory tissue. Professor Carl Philpott, director of medical affairs and research at Fifth Sense, states that while looking at the olfactory tissue microscopically, one will notice the fine hair-like endings of the receptor cells had fallen, thus disabling the cells from receiving olfactory senses from the nose (43). This is a byproduct of the virus interacting with the chemoreceptors in the mucosal membrane and compromising neural sensations. Further histological evidence on peripheral tissue and other mucosal membranes have not been actively reported, thus highlighting limitations to understanding peripheral involvement following viral activity.

According to the American Academy of Otolaryngology—Head and Neck Surgery, otolaryngologists in the U.K have reported that more than two-thirds of confirmed COVID-19 cases in Germany had anosmia (loss of smell) (44). While in South Korea, 30% of patients testing positive have had anosmia as their primary symptom in otherwise mild cases (44). Similar reports have been found globally, as anosmia and ageusia are highly reported as primary symptoms of COVID-19. In another study, patients with confirmed cases of COVID-19 were recruited from 12 European hospitals to investigate olfactory and gaustory dysfunctions (45). Out of the 417 patients studied, between 85.6 and 88% of patients reported these dysfunctions, with significantly more cases in females (45). 44% of the participants also reported their symptoms early in their pathology (45). Physicians state that it's not surprising that the olfactory system might be heavily influenced, given that loss of smell and changes in taste are a rather common complaint of people who get influenza as well (46). Other symptoms may include hyposmia, neuralgia, and skeletal muscular symptoms, however, these symptoms are not considered primary peripheral manifestations due to a lack of supporting evidence. Additionally, a single case suggests a possible association between Guillain-Barré Syndrome (GBS) and SARS-CoV-2 infection (47, 48). A female patient who returned from Wuhan, China, underwent neurologic examination and was diagnosed with GBS while also testing positive for SARS-CoV-2 (48). This was followed with five more cases also displaying COVID-19 with GBS complications (49). GBS is a rare autoimmune disorder that attacks nerves in the PNS and can lead to paresthesia, areflexia, ataxia, and muscle and facial weakness, or paralysis (49). Despite being a unique condition, viral infections can be triggers of GBS, especially when these infections reach a severe state. Primary symptoms of GBS, such as tingling, leg weakness, and facial weakness, were noticed in COVID-19 patients around 5–10 days after the initial common COVID symptoms (49). After a few days, more adverse symptoms such as quadraparesis and paralysis were recognized (49). Moreover, a variant of GBS, known as Miller Fisher Syndrome, has also been reported in two cases of COVID-19 from Madrid, Spain (50). Miller-Fisher Syndrome is characterized by areflexia and paralysis of the eyes and may also be proceeded by a viral agent's neurological activity (51). While still being a rare condition, the association between COVID-19, GBS, and Miller-Fisher Syndrome warrants further epidemiological data to support a causal relationship between them (47).

As far as treatment, research is ongoing with clinical trials still being administered to determine the most viable form of care. Once the virus has started impacting neuronal activity, it becomes very difficult to treat, meaning the best mode of treatment is by preventing or reducing the viral entry. However, The Center of Disease Control recently stated: "There are no drugs or other therapeutics approved by the US Food and Drug Administration to prevent or treat COVID-19” (52). This adds further limitations to addressing PNS neural manifestations of COVID-19. The British Rhinological Society has advised against the use of oral steroids in the treatment of anosmia in COVID-19 patients (53). This precaution comes with the concern that corticosteroid use may increase severity of the pathogen. However, due to the concentrated presence of the coronavirus in the nasal tissues, a nasal spray has been suspected as the best mode in medication delivery (54). Recent reports from Utah have started addressing the efficacy of chlorpheniramine maleate (CPM) combined into a nasal spray to study cultures with SARS-CoV-2 (54). CPM is an antihistamine with previous studies suggesting that it acts well in the antiviral treatment against strains of influenza (54). The initial trials have showed relative success with reduced amounts of the virus being reported *in vivo* following CPM administration (54). However, this requires further testing in order to accurately and effectively treat COVID patients. With more severe patients who may have acquired GBS with COVID-19, intravenous immunoglobulin therapy (IVIG) have been known as the most common treatment for these patients (49). IVIG treatment allows for the administration of healthy antibodies and has been suggested to be taken with antivirals to further combat the COVID like symptoms (49). Plasmapheresis with antiviral treatment has also been suggested as another treatment option to enhance the autoimmune system while reducing viral activity (49). With regards to the two cases with Miller-Fisher Syndrome, the first was treated with IVIG while the second with acetaminophen. After two weeks, it was seen that both patients made a complete neurological recovery, except for primary symptoms of anosmia and ageusia from their COVID-19 diagnosis (50). However, these reports have yet to be confirmed as a definite viable treatment and may not directly lead to every patient's recovery.

To better combat PNS manifestations today, ENT UK released an info-sheet encouraging those who experience sudden loss of smell or taste during the COVID-19 pandemic to assume they have the virus until tests prove negative. The management plan included: self-isolation, avoiding visits to medical professionals or hospitals unless symptoms require proper treatment, and try smell training to improve senses. Furthermore, The AAO-HNS created a web-based reporting tool for any health-care professionals to file reports of patients experiencing anosmia and ageusia related to COVID-19. Until a viable treatment is released to the public, these guidelines will help those with chemosensory dysfunction to be informed and aware of what conditions to follow.

## Avoidance of Detrimental Neurological Sequelae and Multidisciplinary Approach

Following the emergence of more literature findings about the neurological manifestations of COVID-19, an increasing number of studies are now urging clinicians to screen for neurological presentations of this disease (1, 4, 23, 55, 56). As previously mentioned, the symptoms present a neurological pathology, while those with more severe cases have been susceptible to neurological complications. Thus, these studies are urging clinicians to comprehensively assess the neurological symptoms in patients coming into acute healthcare settings, to effectively triage patients, delay diagnosis and misdiagnosis, and prevent transmission of the virus. Although preliminary, studies suggest that surveillance of the neurological manifestations of COVID-19 may have guiding significance for the prevention and treatment of COVID-19 induced respiratory failure and death (4, 55).

Knowledge and clinical guidelines surrounding treatment of COVID-19 is rapidly evolving as more information becomes available. At this time, knowledge surrounding the prevention and treatment of neurological complications of COVID-19 is limited. One study published several clinical guidelines for neurologists treating patients with suspected or confirmed cases of COVID-19(57). These guidelines were pertaining to the management of acute cerebrovascular disease, intracranial infection, and muscle damage as a result of SARS-CoV-2.

In the context of SARS-CoV-2, neurologists may encounter two phenotypes of patients with acute cerebrovascular diseases—acute ischemic stroke patients, and hypertensive patients with increased risk of intracranial hemorrhage. The authors recommend that emergency treatment be jointly offered by neurologists and infectious disease specialists when admitting an acute ischemic stroke patient with suspected or confirmed SARS-CoV-2 diagnosis, and preventive anticoagulation is recommended for ischemic stroke patients with high D-dimer levels (57). Hypertensive patients with SARS-CoV-2 may be particularly challenging to treat, as they may encounter blood pressure fluctuations (since SARS-CoV-2 specifically binds to ACE2 receptors), which may increase their risk of intracranial hemorrhage. Additionally, patients with severe infection may have severe thrombocytopenia, which is another risk factor for cerebral hemorrhage. Per the guidelines, clinicians treating hypertensive patients with SARS-CoV-2 should consider calcium channel blockers, diuretics and other classes of antihypertensive drugs instead of ACE inhibitors or angiotensin II receptor blockers (ARBs) (57).

Further surveillance of intracranial infection for COVID-19 patients would provide further insight in a neurological prognosis. MRI scans (with and without contrast) of the cranium along with lumbar puncture procedures to collect cerebrospinal fluid would highlight such a neuroinvasive association to the pathogen (57). For patients with definitive intracranial infection, suggested treatment strategies are controlling for cerebral edema, treating and preventing seizures, and treating psychotic symptoms (57). Lastly, for patients experiencing symptoms associated with muscle damage, strengthening nutritional support is recommended on top of active treatment for the virus (57).

## Conclusion

In summary, the SARS-CoV-2 pandemic remains one of the greatest threats to public health to date. Although the virus is known to directly invade the lungs, emerging research shows central and peripheral nervous system involvement in the pathology of the disease and clinical worsening in patients. Patients with nervous system involvement as the presenting symptoms in the early stages of infection may be misdiagnosed, and may inadvertently spread the virus. Furthermore, studies suggest that the time it takes for SARS-CoV-2 to advance from initial symptoms, such as coughing, tiredness, and fever, to difficulty breathing, is roughly five days, which is long enough for the virus to enter and damage medullary neurons involved in respiratory control (4, 55). Thus, healthcare providers and neurologists should work closely to monitor the neurological manifestations of the disease and carry a high index of suspicion when evaluating patients in an endemic area. In some cases, tele-neurology as a substitute for face to face interactions with patients to monitor their clinical status may be useful.

Going forward, to elucidate the involvement of the nervous system in the progression of this disease, postmortem examinations of the brain may be a valuable facet in understanding the neurological mechanisms the virus manifests. Currently, few, if any, autopsies are being performed due to the fear of contracting the disease. Furthermore, a more comprehensive neurological follow-up of patients who recovered from the disease may be warranted to screen for long-lasting neurological impacts of the illness and save lives.

## Author Contributions

BF was the primary author contributing to the outline/direction of the paper, literature review, and writing, editing, and overseeing of the paper as a whole including submission process. RJ played a major role in writing and editing the paper. MS, AD, and CC focused their efforts on writing. All authors contributed to the article and approved the submitted version.

## Conflict of Interest

The authors declare that the research was conducted in the absence of any commercial or financial relationships that could be construed as a potential conflict of interest.

## Abbreviations

WHO: World Health Organization
COVID-19: Coronavirus Disease-2019
ICTV: International Committee on Taxonomy of Viruses
SARS-CoV: severe acute respiratory syndrome coronavirus
MERS-CoV: Middle East respiratory syndrome coronavirus.
